# Carbon Nanotube‐Supported Mo, Ni, and Co Nitrides as Stable Catalysts for Levulinic Acid Conversion: Influence of Metal–Nitrogen Interactions and Confinement Effects

**DOI:** 10.1002/open.202500291

**Published:** 2025-07-14

**Authors:** Ivet Pafian, Jorge Noé Díaz de León, Juan Seguel, Néstor Escalona, Gina Pecchi, Carla Herrera, Catherine Sepulveda

**Affiliations:** ^1^ Facultad de Ciencias Químicas Universidad de Concepción Casilla 16C Concepcion 4070371 Chile; ^2^ Centro de Nanociencias y Nanotecnología Universidad Nacional Autónoma de México Carretera Tijuana‐Ensenada Ensenada 22800 Mexico; ^3^ Facultad de Química y de Farmacia Pontificia Universidad Católica de Chile Macul Chile; ^4^ Departamento de Ingeniería Química y Bioprocesos Escuela de Ingeniería Pontificia Universidad Católica de Chile Santiago Chile

**Keywords:** carbon nanotubes, confinement effect, Levulinic acid, nitride phases, oxynitride species

## Abstract

Transition metal nitrides (MxNy, where M = Mo, Ni, or Co) supported on carbon nanotubes (CNTs) are synthesized and evaluated as catalysts for the conversion of levulinic acid at 250 °C and 50 bar H_2_. The catalysts are extensively characterized by N_2_ physisorption, XRD, TEM, FT–IR, H_2_‐TPR, NH_3_–TPD, 2‐propanol conversion, and XPS. Among the series, the Mo_2_N/CNT catalyst exhibits stronger metal–support interaction, smaller particle size, and more pronounced confinement within the CNT structure. This is attributed to the higher Mo–N bond strength compared to Ni–N and Co–N, which also influence the density and strength of surface acid sites. In contrast, the Ni_3_N/CNT catalyst displays the highest catalytic activity and is associated with smaller nitride particles located on the external CNT surface. The Co_4_N/CNT catalyst shows intermediate behavior. Product selectivity is primarily governed by the presence of surface nitride and oxynitride species, rather than the specific nature of the transition metal. These findings highlight the role of metal–support interactions and active phase dispersion in the design of stable, nonnoble metal catalysts for biomass‐derived platform molecule conversions.

## Graphical abstract

Nitride‐phase catalysts (MxNy/CNT) are active in levulinic acid conversions. Ni_3_N/CNT shows the best performance due to hydrogenation‐active sites within and on the surface of CNT graphene layers. The CNT support also enhances the Ni_3_N phase stability, maintaining catalytic activity over multiple recycling tests.

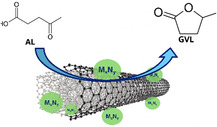

## 1 Introduction

Levulinic acid (LA) is a versatile platform molecule derived from the acid hydrolysis of cellulose and hemicellulose components of lignocellulosic biomass.^[^ ^1^ ^]^ It is soluble in water and polar organic solvents^[^ ^2^, ^3^ ^]^ and can be catalytically converted via hydrogenation, dehydration, and ring‐opening reactions into a variety of value‐added compounds, including γ‐valerolactone (GVL), 1,4‐pentanediol, pentenoic acid, 2‐methyltetrahydrofuran, and pentanoic acid.^[^ ^4^, ^5^, ^6^ ^]^ These products have a wide range of applications as solvents, fuel additives, and intermediates for biofuels and chemicals. The catalytic hydrogenation of LA has been widely investigated using both homogeneous and heterogeneous catalysts.^[^ ^7^, ^8^, ^9^ ^]^ Heterogeneous systems based on noble metals such as Ru, Pd, and Pt exhibit high efficiency,^[^ ^9^, ^10^, ^11^ ^]^ but their cost and scarcity have motivated the search for nonnoble metal alternatives, including Ni‐, Co‐, and Cu‐based catalysts.^[^ ^12^, ^13^, ^14^, ^15^ ^]^ Despite their promising activity, nonnoble metal catalysts often suffer from leaching, particle sintering, and oxidation state changes under liquid‐phase conditions, which limit their stability and practical applicability.^[^ ^6^, ^16^, ^17^ ^]^

Transition metal nitrides (MxNy) have emerged as robust and active catalysts with enhanced resistance to oxidation compared to their metallic counterparts. Their superior performance is attributed to their ability to store hydrogen in interstitial sites, modulate the electronic density of the metal center, and facilitate selective C=O bond hydrogenation.^[^ ^18^ ^]^ The incorporation of nitrogen also leads to lattice expansion and defect formation, enhancing oxygen removal during hydroprocessing reactions.^[^ ^19^ ^]^ These features make nitrides comparable in activity to noble metal catalysts such as Pd and Pt.^[^ ^20^ ^]^ Mo_2_N‐based catalysts, for instance, have shown excellent performance in hydrodeoxygenation of guaiacol,^[^ ^21^, ^22^, ^23^, ^24^ ^]^ while Ni_3_N and Co_4_N catalysts have demonstrated improved resistance to coking and deactivation.^[^ ^25^ ^]^

Carbon‐based materials, particularly carbon nanotubes (CNTs), have gained attention as catalyst supports due to their high surface area, thermal stability, and low tendency to promote coke formation.^[^ ^26^, ^27^, ^28^, ^29^ ^]^ In hydrotreatment reactions, CNTs have been shown to stabilize metal nanoparticles, enhance dispersion, and improve catalyst durability.^[^ ^30^, ^31^, ^32^, ^33^ ^]^ For the hydroprocessing of anisole in gas phase on Mo/CNT‐supported catalysts at 300–400 °C and 80 bar of H_2_ pressure,^[^ ^34^ ^]^ the authors proposed that Mo species confined within the nanotubes promotes the strong interaction of the metal with the support, increasing the resistant to sintering and contributing to long catalyst lifetime. Liu et al.^[^ ^35^ ^]^ on Ni/CNTs and NiCu/CNTs catalysts report the conversion of furfural at 130 °C, 40 bar of H_2_ and ethanol as solvent with large selectivity (85 % to 90%) to tetrahydrofurfuryl alcohol (THFA). The higher conversion of furfural and enhanced selectivity to THFA of the Ni/CNTs and NiCu/CNTs catalysts compared with their respective Ni and NiCu supported on MgO, γ‐Al_2_O_3_, TiO_2_, and ZrO_2_ counterparts is attributed to the favorable structure of CNTs, promoting the confinement and activation of the metallic components and improving their catalytic performance. Bimetallic MNi/CNT (M=Co, Cu, Fe)‐supported catalysts tested in the glycerol steam reforming^[^ ^36^ ^]^ indicates that the CoNi/CNTs and Ni/CNTs catalysts display better properties in both, the antisintering and coke resistance, making the reaction more selective to the hydrogen production enhancing the WGSR. The activity of the surface‐oxidized Co/CNTs catalysts in the Fischer–Tropsch reaction^[^ ^37^ ^]^ showed that the large catalytic activity of Co/CNTs is attributed to a smaller particle size of Co confinement in the CNTs structure and to the hydrogen spillover effect by the quinone surface functional groups of the CNTs.

The efficient conversion of LA into valuable chemicals requires catalysts that integrate multiple functionalities: acidic sites for dehydration, metallic sites for hydrogenation, and oxygen‐deficient structures for partial deoxygenation and ring opening. In this context, transition metal nitrides supported on CNTs represent a promising platform due to their combined acidity, redox stability, and confinement effects.

In this work, we report the synthesis, characterization, and catalytic evaluation of Mo_2_N, Ni_3_N, and Co_4_N catalysts supported on CNTs for LA conversion. By correlating the structural, textural, and surface properties with catalytic performance, this study reveals the impact of metal–nitrogen interactions and CNT confinement on catalyst activity and stability. To the best of our knowledge, this is the first comparative study exploring CNT‐supported transition metal nitrides for LA conversion, providing new insights into structure–activity relationships in nonnoble metal catalytic systems.

## 2 Results and Discussion

### 2.1 Characterization

**Figure 1:**
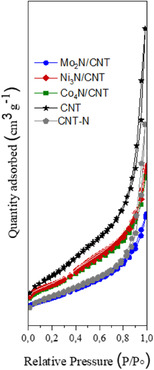
Adsorption–desorption isotherms of N_2_ at 77 K to CNT, CNT‐N and Mo_2_N/CNT, Ni_3_N/CNT, and Co_4_N/CNT catalysts.

**Figure** **1** displays the N_2_ adsorption–desorption isotherms at 77 K for CNT, CNT‐N, and the Mo_2_N/CNT, Ni_3_N/CNT, and Co_4_N/CNT catalysts. All the samples exhibit type IV isotherms with H3‐type hysteresis loops, indicative of mesoporous structures, in accordance with the IUPAC classification.^[^ ^38^, ^39^ ^]^

Textural properties and metal loading are summarized in **Table** **1**.

**Table 1:** Metal composition, textural properties, and catalytic activity results of the fresh and used catalysts.

	SBET [m^2^ g^−1^]	Vp [cm^3^ g^−1^]	wt%	Xt [%]	rs × 102 mol gcat^−1^ min^−1^
CNT	241	0.43	–	–	–
CNT‐N	174	0.33	–	–	–
Fresh					
Mo_2_N/CNT	206	0.24	9.5 (5.0)	22	4.6
Ni_3_N/CNT	237	0.33	6.1 (5.0)	70	9.5
Co_4_N/CNT	235	0.32	8.0 (5.0)	40	7.4
Postreaction					
Mo_2_N/CNT	119	0.23	5.6	–	–
Ni_3_N/CNT	120	0.35	4.8	–	–
Co_4_N/CNT	139	0.35	3.8	–	–
Recycles					
Ni_3_N/CNT(R1)	110	0.36	4.4	50	5.8
Ni_3_N/CNT(R2)	108	0.38	4.0	43	5.5

The decrease in BET surface area observed for CNT‐N compared to pristine CNT is attributed to the partial degradation or shortening of the nanotubes during nitridation, as confirmed by transmission electron microscopy (TEM) analysis. The experimentally determined metal loadings (AAS) slightly exceed the nominal 5 wt%, likely due to CNT mass loss during NH_3_ treatment, resulting in apparent enrichment of metal content.

**Figure 2:**
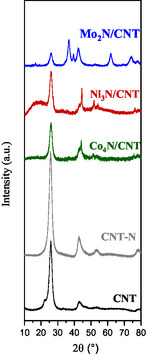
XRD of CNT, CNT‐N and Mo_2_N/CNT, Ni_3_N/CNT, and Co_4_N/CNT catalysts.

XRD patterns (**Figure** **2**) confirm the successful formation of metal nitride phases. The diffractograms were compared with patterns of CNT (JCPDS‐ICDD, 75‐1621), metal nitrides γ‐Mo_2_N (JCPDS‐ICDD, 25‐1366), Ni_3_N (JCPDS‐ICDD, 00‐010‐0280), Co_4_N (JCPDS‐ICDD, 41‐0943), and metallic Ni (JCPDS‐ICDD, 03‐065‐0380). CNT and CNT‐N show characteristic peaks at 2θ = 25.7° and 42.8°, corresponding to the graphitic structure.

Additional peaks at 2θ = 53.1° and 77.9° indicate the presence of the graphene phase,^[^ ^40^ ^]^ suggesting that the C—C bonds remain dominant over the C—N formation during nitridation. Mo_2_N/CNT exhibits peaks corresponding to γ‐Mo_2_N and β‐Mo_2_N, with no detectable Mo oxide signals, confirming complete nitridation. Ni_3_N/CNT shows Ni_3_N and metallic Ni° reflections, consistent with partial decomposition during nitridation.^[^ ^41^ ^]^ Co_4_N/CNT shows well‐defined Co_4_N reflections, with no evidence of metallic Co or oxide species.

Thermogravimetric analysis in oxidizing conditions (Figure S1, Supporting Information) demonstrates the increased thermal stability of CNT‐N compared to untreated CNT, confirming the structural integrity after nitridation. All metal nitride catalysts are thermally stable above 400 °C and suitable for catalytic applications.

**Figure 3:**
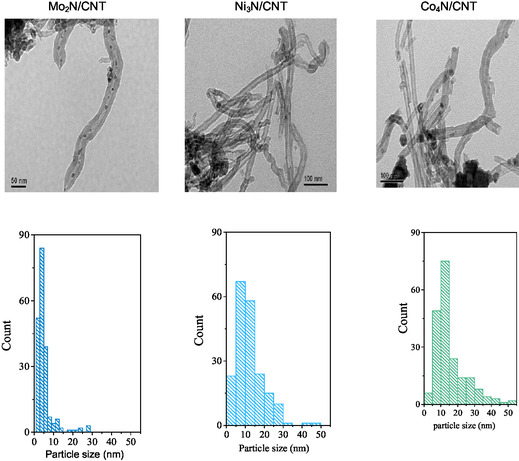
TEM micrographs and the corresponding histograms of the particle size distribution of Mo_2_N/CNT, Ni_3_N/CNT, and Co_4_N/CNT catalysts.

TEM images and particle size histograms (**Figure** **3**) show that Mo_2_N particles are primarily confined within the CNT channels (≈5.3 nm), while Ni_3_N and Co_4_N are more externally dispersed with larger particle sizes (≈12.2 nm and 15.7 nm, respectively). This confinement effect may influence both stability and accessibility of active sites.

**Figure 4:**
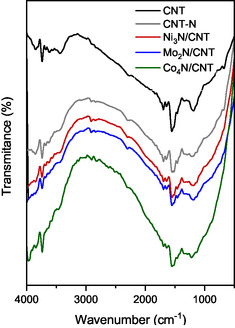
FTIR spectra of the CNT, CNT‐N and Mo_2_N/CNT, Ni_3_N/CNT, Co_4_N/CNT catalysts.

FTIR spectra (**Figure** **4**) show O–H stretching at 3735 cm^−^^1^ and 3436 cm^−^^1^ in CNT and CNT‐N, related to ambient moisture, respectively. The characteristic C=C vibration of graphene appears at 1550 cm^−^^1^. Nitrided samples show new bands at 1646 cm^−^^1^ and 1186 cm^−^^1^, attributed to the N–H and C–N bonds, respectively,^[^ ^42^, ^43^, ^44^ ^]^ confirming nitrogen incorporation. Mo_2_N/CNT and Ni_3_N/CNT exhibit moderate intensities for these bands, while Co_4_N/CNT shows stronger absorption, suggesting more extensive nitride phase exposure.

**Figure 5:**
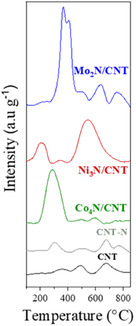
H_2_‐TPR of CNT, CNT‐N and Mo_2_N/CNT, Ni_3_N/CNT, and Co_4_N/CNT catalysts.

For the nitride‐metal catalysts, temperature‐programmed reduction (H_2_‐TPR) profiles (**Figure** **5**) show weak reduction signals for CNT and CNT‐N at ∼300 °C and ≈770 °C, attributed to surface and internal C=N bond reduction.

The difference in temperature for the appearance of the reduction peaks for CNT and CNT‐N is attributed to the environment of the C=N bonds, being the surface bonds reduced at lower temperature, and at higher temperatures, the C=N bonds inside the CNT. Mo_2_N/CNT displays two overlapping peaks at 366 °C and 405 °C associated with NH_x_ reduction in γ‐ and β‐Mo_2_N phases, and additional peaks at 637 °C and 758 °C related to bulk Mo_2_N reduction and confined C = N bond reduction.^[^ ^45^ ^]^ Ni_3_N/CNT shows surface NH_x_ reduction at 204 °C and Ni_3_N to Ni° reduction at 544 °C.^[^ ^41^ ^]^ Co_4_N/CNT presents a single reduction peak at 285 °C, associated with Co_4_N to Co° transformation.^[^ ^46^ ^]^

**Figure 6:**
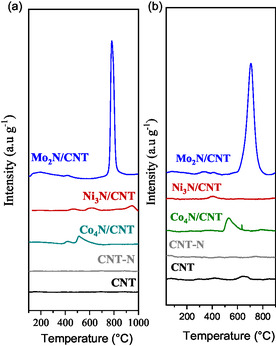
a) NH_3_‐TPD, b) CO_2_‐TPD of CNT, CNT‐N, and Mo_2_N/CNT, Ni_3_N/CNT, and Co_4_N/CNT catalysts.

In **Figure** **6**, the NH_3_–TPD profiles indicate the strength of the acid sites classified as weak (<300 °C), medium (300 °C < T < 500 °C) and strong (>500 °C).^[^ ^47^ ^]^

NH_3_–TPD profiles (Figure 6a) show negligible ammonia desorption for CNT and CNT‐N, indicating an absence of significant acidity. Mo_2_N/CNT shows a strong desorption peak at 781 °C, confirming the presence of strong acid sites. Ni_3_N/CNT and Co_4_N/CNT exhibit broad, low‐intensity peaks, suggesting fewer and weaker acid sites.

CO_2_–TPD results (Figure 6b) follow a similar trend. Mo_2_N/CNT shows a prominent peak at ∼800 °C, attributed to strong basic sites. Co_4_N/CNT exhibits weak basicity, while Ni_3_N/CNT shows negligible CO_2_ desorption, indicating limited or undetectable basic sites. These results suggest that the acid–base properties depend more strongly on the metal–nitrogen bond than on the specific nitride phase. Mo_2_N, with higher Mo–N interaction, exhibits stronger acid and basic site densities compared to Ni_3_N and Co_4_N.

To identify Lewis and Brønsted sites, the catalytic conversion of 2‐propanol was carried out as an indirect method to evaluate the acid properties.

The dehydration reaction of 2‐propanol to propylene occurs on Lewis acid sites and the dehydrogenation of 2‐propanol to acetone occurs on the Brønsted acid sites;^[^ ^48^ ^]^ therefore, the product distribution of this catalytic reaction can be related to the amount of Lewis and Brønsted sites. 2‐propanol conversion (Figure S3, Supporting Information) was used to distinguish Brønsted and Lewis acidity. Propylene formation (dehydration) indicates Lewis sites, while acetone (dehydrogenation) suggests Brønsted sites. Mo_2_N/CNT shows a higher proportion of Lewis sites, while Ni_3_N/CNT and Co_4_N/CNT exhibit a more balanced acidity profile.

**Figure 7:**
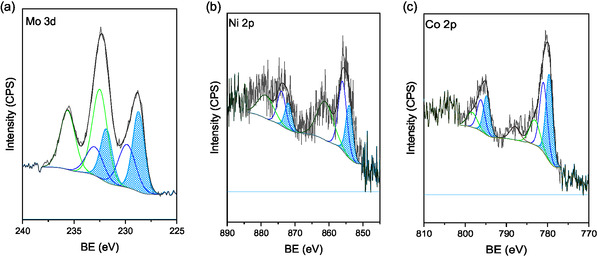
XP spectra of a) Mo 3d_5/2_, b) Ni 2p_3/2_, and c) Co 2p_3/2_ for the Mo_2_N/CNT, Ni_3_N/CNT, and Co_4_N/CNT catalysts.

The XP spectra of the Mo 3d_5/2_, Ni 2p_3/2_, and Co 2p_3/2_ core‐level spectra are shown in **Figure** **7**, the C1s and N1s in the supplementary information, and the binding energy (BE) values and surface distribution in **Table** **2**. The deconvoluted Mo 3d_5/2_ X‐ray photoelectron spectroscopy (XPS) (Figure 7a) shows several oxidation states ranging from Mo^2+^ to Mo^6+^ at 228.7 eV assigned to surface Mo_2_N, with Mo^*δ*+^ (2 < *δ* < 4) at 229.9 eV to Mo^4+^ and at 232.5 eV to Mo^6+^ in molybdenum oxynitrides.^[^ ^20^, ^23^, ^49^ ^]^ For Ni_3_N/CNT, the doublet for Ni 2p_3/2_ at 854.0 and 856.0 eV in Figure 7b to surface nickel nitride and oxynitride.^[^ ^50^ ^]^ For Co_4_N/CNT, Figure 7c shows the Co 2p_3/2_ signal for BE at 779.6 eV attributed to cobalt–nitride species, and at 781.0 eV and 783.1 eV to Co^3+^ of cobalt oxynitrides.^[^ ^23^, ^51^ ^]^

**Table 2:** Binding energies of Mo 3d_5/2_, Ni 2p_3/2_, Co 2p_3/2_, and N 1s for the Mo_2_N/CNT, Ni_3_N/CNT, and Co_4_N/CNT catalysts. Surface extent in parenthesis.

	BE, eV	
Mo_2_N/CNT	Mo 3d_5/2_ 228.7 (31) 229.9 (25) 232.5 (44)	N 1s 395.0 397.8
Ni_3_N/CNT	Ni 2p_3/2_ 854.0 (39) 856.0 (61)	N 1s n.d.
Co_4_N/CNT	Co 2p_3/2_ 779.6 (42) 781.0 (39) 783.1 (19)	N 1s n.d.

n.d.: not detected.

The surface extent of the nitride phase (area under curve highlighted in blue color in Fig. a, b, and c), indicates in Table 2, in line with TEM results, a surface of 31% of Mo_2_N (228.7 eV), 39% of Ni_3_N (854.0 eV), and 42% of Co_4_N (779.6 eV).

The similar surface oxynitride phase contributions (≈70%) of the Mo_2_N/CNT, Ni_3_N/CNT, and Co_4_N/CNT catalysts are in line with Tyrone Ghampson et al.^[^ ^23^ ^]^ who reported protected Mo_2_N supported on the Al_2_O_3_ and MCM‐41 surface by reduction and passivated treatments. Therefore, the large stability of the surface nitride phase in the Mo_2_N/CNT, Ni_3_N/CNT, and Co_4_N/CNT catalysts can be attributed to the confinement effect of the CNT. Regarding the presence of the surface N, the XPS of N1s (Figure S4, Supporting Information) indicates only surface contributions of nitrogen assigned to the surface Mo_2_N species at 394.9 eV and 397.5 eV,^[^ ^52^, ^53^ ^]^ not detected for Ni_3_N/CNT and Co_4_N/CNT. The XP spectra of C1s (Figure S5, Supporting Information) display the expected signals at 284.3 eV of the C=C bond of the graphene sheets, 285.1 eV of the C–C bond, 286.0 and 287.2 eV of the C–O bonds, C–H, and C–N bonds, 289.0 eV C = O and at 291.0 eV the O–C=O bonds characteristic of carboxylic acids, esters, and other organic functional groups^[^ ^54^, ^55^, ^56^, ^57^ ^]^ in the surface of the Mo_2_N/CNT, Ni_3_N/CNT, and Co_4_N/CNT catalysts.

## 3 Catalytic Activity

### 3.1 Fresh Catalysts

The catalytic performance of Mo_2_N/CNT, Ni_3_N/CNT, and Co_4_N/CNT catalysts in the conversion of LA was evaluated in terms of total conversion (X_t_) and initial reaction rate (r_s_), as summarized in Table 1 and Figure S6, Supporting Information. The blank experiment with CNT‐N showed no detectable LA conversion, confirming the necessity of the active metal nitride phase.

The largest conversion level obtained for Ni_3_N/CNT, the same trend of initial rate, can be explained considering the distribution of the active phase inside and outside graphene sheets of the CNT, allowing accessibility of LA, promoting the catalytic activity. To explain the lowest catalytic performance of Mo_2_N/CNT, it is proposed that the confinement effect favors the stability of the active phase, promoting a smaller particle size, and hinders the access of LA to the active nitride sites. The catalytic performance of Co_4_N/CNT is attributed to the surface aggregates of large particle size, decreasing the surface availability of the active sites. This catalytic performance indicates that under the used reaction conditions, the active sites in the conversion of LA are the surface nitride species with not a large effect of the particle size.

**Figure 8:**
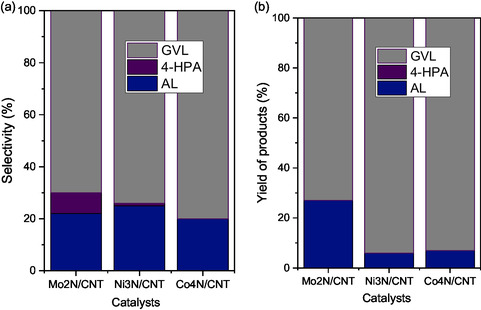
a) Selectivity at 10% of LA conversion. b) Yield of products at 240 min of reaction for the Mo_2_N/CNT, Ni_3_N/CNT, Co_4_N/CNT catalysts.

Product distribution analysis (**Figure** **8**a) reveals that the main reaction intermediates are angelica lactone (AL) and 4‐hydroxypentanoic acid (4‐HPA), with GVL as the principal final product. These results are consistent with reported pathways^[^ ^4^, ^5^, ^58^ ^]^ in which LA conversion proceeds via parallel dehydration (to AL) and hydrogenation (to 4‐HPA) routes, followed by hydrogenation or dehydration to GVL.

Selectivity data at 10% LA conversion suggest that all catalysts initially favor the formation of AL via acid‐catalyzed dehydration, followed by its hydrogenation to GVL. The Mo_2_N/CNT catalyst exhibited a higher proportion of 4‐HPA, indicative of its strong hydrogenation capability. However, the slower overall kinetics likely result from limited accessibility to active sites due to confinement effects.^[^ ^59^ ^]^ Final product yields at 240 min (Figure 8b) show GVL as the dominant product and AL as the only remaining intermediate. This behavior correlates with the ∼70% surface oxynitride species identified by XPS for all catalysts,^[^ ^60^, ^61^ ^]^ highlighting the importance of the Brønsted and Lewis acid site strength in determining the rate and extent of the LA conversion. These findings are supported by the 2‐propanol dehydration results, which confirm the presence of acid sites, with a slight Brønsted acidity predominance in Ni_3_N/CNT and Co_4_N/CNT. Overall, the superior GVL yield (∼90%) for Ni_3_N/CNT and Co_4_N/CNT compared to Mo_2_N/CNT (≈70%) demonstrates the critical role of accessible hydrogenation sites and surface composition. The catalytic behavior observed is comparable to that of the noble metal‐based systems and can be explained by the interaction between metal *d* orbitals and nitrogen *sp* orbitals, leading to stabilized metal/nonmetal bonds and modulated electronic properties.^[^ ^62^ ^]^

### 3.2 Characterization Post Reaction

**Figure 9:**
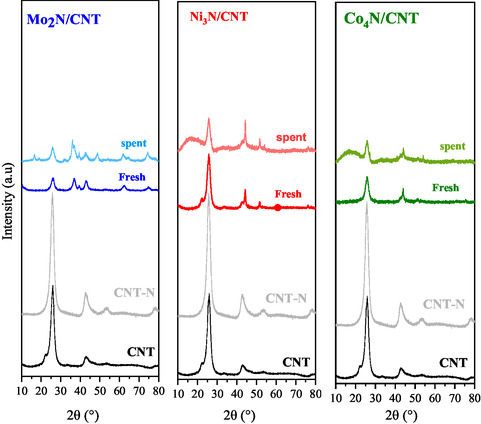
XRD of CNT, CNT‐N and Mo_2_N/CNT, Ni_3_N/CNT, and Co_4_N/CNT catalysts: fresh and post reaction.

Postreaction XRD patterns (**Figure** **9**) confirm the structural stability of the nitride phases. For Mo_2_N/CNT, the characteristic reflections of γ‐Mo_2_N and β‐Mo_2_N remain, along with the appearance of a peak at 2θ = 48.8°, corresponding to MoO_2_, suggesting partial surface oxidation. For Ni_3_N/CNT and Co_4_N/CNT, the respective reflections for Ni_3_N/Ni° and Co_4_N remain unchanged, confirming high‐phase stability during reaction.

AAS results (Table 1) indicate metal leaching during the catalytic tests, with the highest loss for Co (53%), followed by Mo (41%), and Ni (21%). The lower leaching observed for Ni_3_N/CNT supports the hypothesis of more stable metal–support interactions and better nitride phase anchoring in this system.

### 3.3 Catalysts Recycling

**Figure 10:**
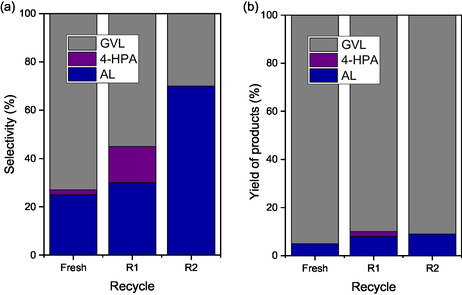
a) Selectivity at 10 % of LA conversion. b) Yield of products at 240 min of reaction of fresh, R1 and R2 of Ni_3_N/CNT catalyst.

**Figure 11:**
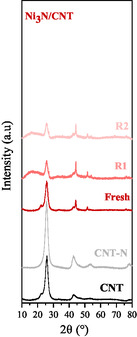
XRD of CNT, CNT‐N, and fresh (R1) and second (R2) recycled Ni_3_N/CNT catalyst.

Based on its superior catalytic performance and lower metal loss, Ni_3_N/CNT was selected for recycling tests. After each reaction, the catalyst was recovered by vacuum filtration, washed with dioxane, and dried at 120 °C. The catalytic data for fresh catalyst and after the first (R1) and second (R2) reuse cycles are presented in Table 1 and Figure S7, Supporting Information. A progressive decline in X_t_ was observed from 70% (fresh) to 50% (R1) and 43% (R2). The initial rate dropped by ≈40% after the first cycle and remained relatively constant thereafter. Selectivity trends (**Figure** **10**a) show increased AL and reduced GVL formation in the recycled samples, suggesting a loss of hydrogenation functionality. However, product yields at 240 min (Figure 10b) remain consistent between R1 and R2, indicating that the nature of the active sites remains unchanged. XRD analysis of fresh and recycled Ni_3_N/CNT samples (**Figure** **11**) confirms the structural integrity of the Ni_3_N phase throughout both recycling steps. The diffraction patterns show consistent peak intensity and width, indicating that the phase crystalline is preserved. These findings suggest that the decline in the catalytic performance is due to a decrease in the number of active sites, primarily from Ni leaching, rather than deactivation or structural degradation of the nitride phase.

## 4 Conclusion

CNT‐supported metal nitrides Mo_2_N, Ni_3_N, and Co_4_N were successfully synthesized and evaluated as catalysts for the conversion of LA under mild hydrogenation conditions (250 °C, 50 bar H_2_). Comprehensive characterization confirmed the structural integrity of the CNT support after nitridation and revealed significant differences in metal–support interactions, particle size, and dispersion among the catalysts. The Mo_2_N/CNT catalyst exhibited a pronounced confinement effect, with small Mo_2_N nanoparticles located within the CNT channels. This led to enhanced structural stability, strong metal–nitrogen interactions, and higher acid–base site density. However, this confinement also restricted access to the active phase, resulting in lower catalytic activity. In contrast, the Ni_3_N/CNT catalyst showed the highest catalytic performance, attributed to its smaller particle size, external dispersion of the active phase, and better accessibility of the hydrogenation sites. Co_4_N/CNT displayed intermediate behavior in terms of both structure and activity. All three catalysts showed similar product selectivity, with GVL as the main product, and surface oxynitride species identified as the key contributors to activity, rather than the specific nature of the metal. This suggests that the surface nitride/oxynitride layer is the principal active phase in LA conversion. Additionally, catalyst recycling tests demonstrated that the Ni_3_N/CNT catalyst retained phase stability across multiple cycles, with performance loss primarily due to metal leaching, not deactivation of the active sites.

Overall, this study highlights the potential of the CNT‐supported transition metal nitrides as stable, nonnoble metal catalysts for biomass‐derived platform molecule upgrading. The role of the metal–nitrogen interactions and confinement effects in determining the catalyst performance provides valuable insights for the design of advanced catalytic systems for sustainable chemical transformations.

## 5 Experimental Section

5.1

5.1.1

##### Synthesis of Catalysts

The catalysts were prepared by incipient wetness impregnation to achieve a nominal metal loading of 5 wt%, using aqueous solutions of the corresponding metal nitrate precursors. After impregnation, the samples were aged at room temperature for 48 h, dried at 110 °C for 12 h, and subjected to a nitriding treatment under NH_3_ gas. The nitriding procedure was carried out in three heating stages: from room temperature to 300 °C at 10 °C min^−^^1^, from 300 °C to 500 °C at 0.6 °C min^−^^1^, and from 500 °C to 700 °C at 2 °C min^−^^1^, holding at the final temperature for 2 h. The samples were then cooled under NH_3_ flow to room temperature and labeled as Mo/CNT, Ni/CNT, and Co/CNT, respectively. As a control, commercial pristine CNTs (CheapTubes) were also subjected to the same nitriding treatment and labeled CNT‐N.

##### Catalysts Characterization

The bulk composition of the Mo, Ni, Co was determined by AAS in a Thermo Scientific instrument model ICE Series 3000. X‐ray powder diffraction analysis was recorded on a Bruker D4 Endeavor AXS diffractometer at 40 kV and 20 mA, equipped with CuKα1 radiation (*λ* = 1.5418 Å). The values of 2θ were scanned from 10 to 80° at 0.02 per step. BET Surface area (S_BET_) and textural properties were determined by N_2_ physisorption at 77 K, using a Micromeritics Tristar II 3020 instrument. H_2_‐TPR was obtained using Micromeritics TPD/TPR 2900 equipment equipped with a thermal conductivity detector. In each experiment, 0.050 g of the sample was heated under 5% H_2_/Ar with a flow of 50 mL min^−1^. The sample was heated at a rate of 10 °C min^−1^, from room temperature to 900 °C. Acid properties were determined by NH_3_‐TPD in a Micromeritics AutoChem II instrument equipped with a TCD. A sample of 50 mg was placed in a U‐shaped quartz reactor and pretreated in He flow at 50 mL min^−1^, from room temperature to 110 °C (10 °C min^−1^) for 30 min. The sample was cooled to 42 °C and saturated with NH_3_ at a flow rate of 10 mL min^−1^ of for 10 min. To remove physisorbed NH_3_, the sample was purged with a flow rate of 50 mL min^−1^ of Ar at 75 °C per 1 h. The amount of NH_3_ desorbed was calculated from the area under the curve using ammonia TPD analytical areas. The basicity of the calcined and reduced perovskite catalysts was determined by temperature‐programmed desorption of carbon dioxide (CO_2_‐TPD) using the same apparatus described for NH_3_‐TPD. The sample was first dried under He (50 mL min^−1^) at 100 °C for 1 min to clean the surface. The sample was then saturated with CO_2_ with a flow of 50 mL min^−1^ at 100 °C for 30 min, and subsequently cooled to room temperature under He. Once the TCD baseline was restored, CO_2_‐TPD was performed at a heating rate of 10 °C min^−1^ up to 850 °C. TEM images were acquired using a JEOL Model JEM‐1200 EXII microscope. Samples were ground and dispersed in methanol and transferred to a copper grid (methanol dispersion method). To obtain the nitride particle size, histogram plots for over 300 particles of the MxNy/CNTs catalysts were depicted using the software Image Tool 3.0 software. XPS was recorded on a VG Escalab 200 R electron spectrometer using a Mg Kα (1253.6 eV) photon source. BE were referenced to the C1s level of carbon support at 284.8 eV. An estimated error of ± 0.1 eV can be assumed for all the measurements. The intensities of the peaks were calculated from the respective peak areas after background subtraction and spectrum fitting by the standard computer‐based statistical analysis, which included fitting the experimental spectra.

##### Catalytic Tests

The catalytic conversion of LA was carried out in a Batch Parr 4848 reactor under kinetic control. In each experiment, 4 mL of LA (0.232 mol L^−1^), 80 mL of 1.4‐dioxane (solvent), and 700 μL of n‐hexadecane, as an internal standard, were added to the vessel reactor. After adding the catalysts (0.250 g), the reactor was purged with He flow for 15 min to remove the oxygen content inside the reactor. Still, in the atmosphere, the reactor was heated up to the reaction temperature of 250 °C under stirring at 600 rpm to minimize internal and external mass transfer limitations.^[^ ^63^ ^]^ H_2_ was adjusted to 50 bar of pressure, which was kept constant during the experiment, and aliquots were periodically withdrawn during the reaction. The reaction products were analyzed by gas chromatography (PerkinElmer, Clarus 400) with a flame‐ionization detector and a Cp‐Sil 5 Agilent column (30 m × 0.53 mm × 1.0 μm film thickness). The products were also identified by their column retention time in comparison with available standards. The conversion of LA and selectivity (calculated at 10 % of LA conversion) is defined as follows in Equation (1) and (2)

(1) $$Xt=\frac{\text{inicial mol LA‐final mol LA}}{\text{initial mol of LA }}\;\times 100\;$$

(2)

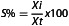

Where Xt is the conversion of LA and Xi is the formation of each product.

The specific rate, r_s_ (molg_cat_^−1^s^−1^) was calculated from the initial slope of the plot of LA conversion as a function of time, according to Equation (3).

(3) $$r_{0}=\frac{\left(bxn\right)}{m}$$

Where *b* is the initial slope of conversion vs time plot (s^−1^), *n* is the initial moles of LA in the solution (mol), and *m* the mass of catalyst (g).

## Conflict of Interest

The authors declare no conflict of interest.

## Supporting information

Supplementary Material

## Data Availability

The data that support the findings of this study are available from the corresponding author upon reasonable request.
