# Supplementary material for: Carbon Nanotube‐Supported Mo, Ni, and Co Nitrides as Stable Catalysts for Levulinic Acid Conversion: Influence of Metal–Nitrogen Interactions and Confinement Effects
Source: ChemistryOpen. 2025 Jul 14;14(11):e202500291. doi: 10.1002/open.202500291 (PMC12598831; doi:10.1002/open.202500291)
Supplement: Supplementary file 1 — Supplementary Material [file OPEN-14-e202500291-s001.pdf]

CNT-supported Mo, Ni and Co nitrides catalysts for levulinic acid conversion

I. Pafian [a], J. Noé Díaz de León [b], J. Seguel [d], N. Escalona [c,d], Gina Pecchi[a], Carla Herrera[a], Catherine Sepulveda[a]*

[a] I. Pafian, Dra. C. Herrera, Dra. G. Pecchi and Dra. C. Sepulveda
Facultad de Ciencias Químicas, Casilla 160C, Universidad de Concepción, 4070371 Chile
E-mail: cathsepulveda@udec.cl

[b] Dr. J. Noé Díaz de León
Centro de Nanociencias y Nanotecnología, Universidad Nacional Autónoma de México, Carretera Tijuana-Ensenada, Ensenada 22800, Mexico

[c] Dr. N. Escalona
Departamento de Ingeniería Química y Bioprocesos
Escuela de Ingeniería Pontificia Universidad Católica de Chile (Chile)

[d] Dr. N. Escalona
Facultad de Química y de Farmacia Pontificia Universidad Católica de Chile (Chile)

Figure S1: TGA of the CNT, CNT-N and Mo_2N/CNT, Ni_3N/CNT, Co_4N/CNT catalysts.

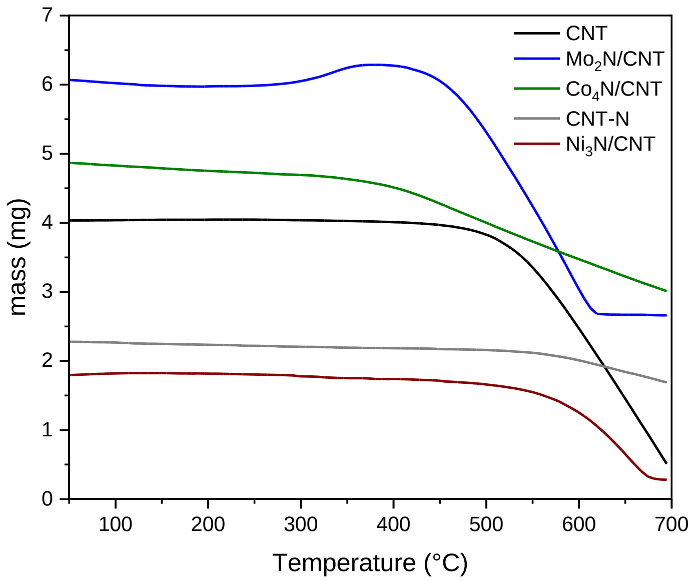

Figure S2: micrographs of CNT and CNT-N

CNT pristine	CNT-N
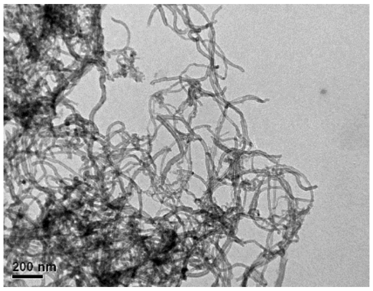	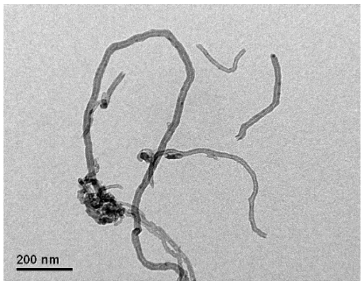
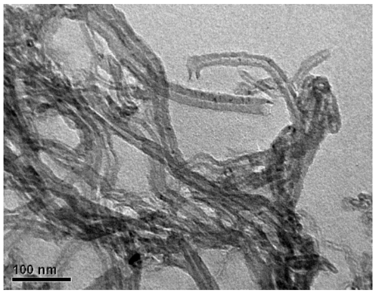	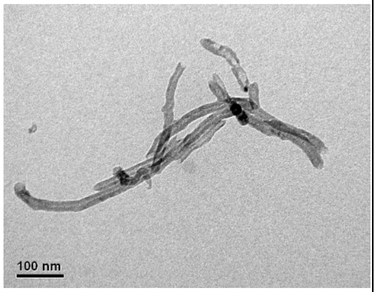

Figure S3. Product distribution of the dehydrogenation of 2-propanol reaction for Mo2N/CNT, Ni3N/CNT and Co4N/CNT catalysts.

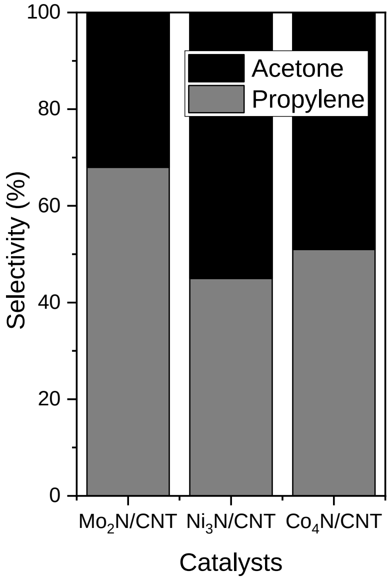

Figure S4. N 1s spectra for Mo2N/CNT, Ni3N/CNT and Co4N/CNT catalysts.

Fig S4 a	Fig S4 b	Fig S4 c
Mo_2N/CNT	Ni_3N/CNT	Co_4N/CNT
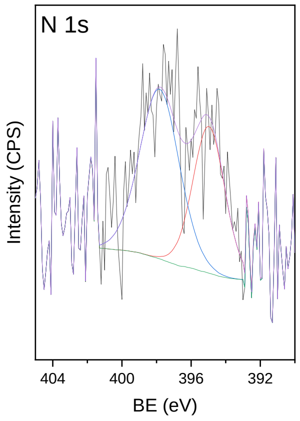	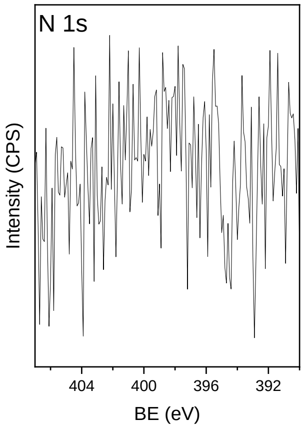	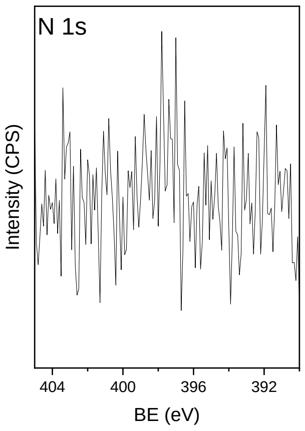

Figure S5. C 1s spectra for Mo_2N/CNT, Ni_3N/CNT and Co_4N/CNT catalysts.

Fig S5 a	Fig S5 b	Fig S5 c
Mo_2N/CNT	Ni_3N/CNT	Co_4N/CNT
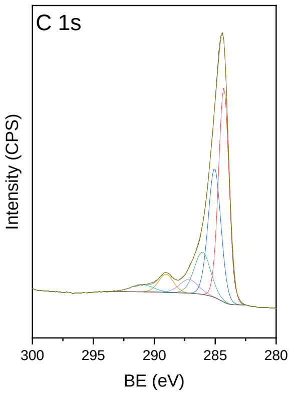	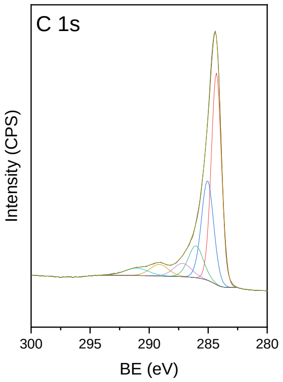	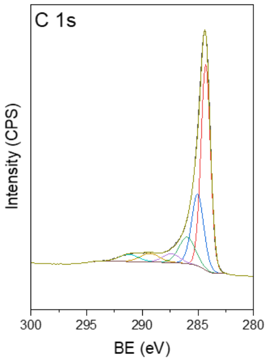

Figure S6. LA conversion upon time for Mo_2N/CNT, Ni_3N/CNT and Co_4N/CNT catalysts.

Mo_2N/CNT	Ni_3N/CNT	Co_4N/CNT
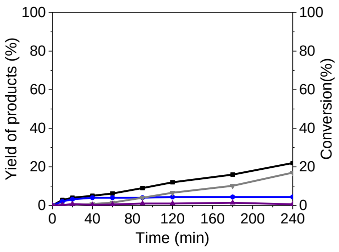	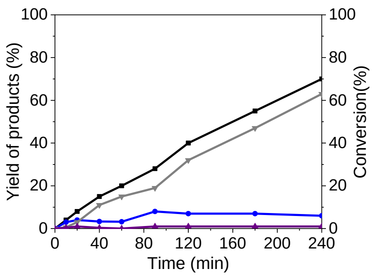	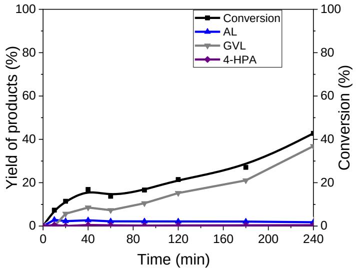

Figure S7. LA conversion upon time for fresh and post reaction Ni_3N/CNT catalyst.

Ni_3N/CNT (fresh)	Ni_3N/CNT (recycle 1)	Ni_3N/CNT (recycle 2)
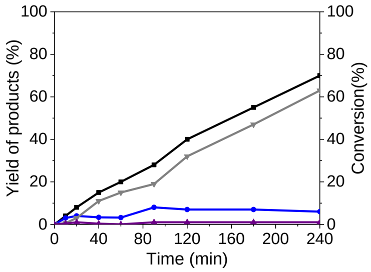	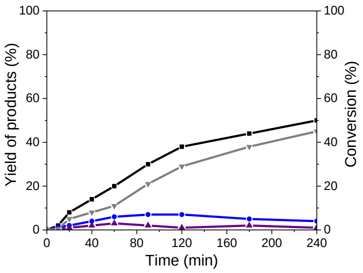	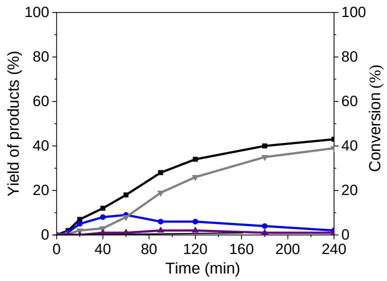
